# More Croup

**Published:** 1889-06

**Authors:** W. S. Gee


### MORE "CROUP."

The controversy regarding the treatment of croup has been in the main interesting, and to some degree, profitable. My good old friend, Dr. Wells, would have placed himself in a better position, if he had adhered to the law by telling us WHEN to give Acon, Hepar, and Spongia.

Surely something more than the name "croup" is necessary to make a homœopathic prescription.

No one is more conscious of this fact, probably, than the learned doctor, but he certainly talks pathology, diagnosis, and remedy in such close "succession" that one may easily infer a leaning to "pathological prescribing."

The statement that so many "hundred cases were thus treated without a loss," is the argument used to induce people to take a patent nostrum, and is not very convincing to a physician who is earnestly seeking for undoubted evidence that the *recoveries were due to the prescribed remedy.*

It is a very doubtful question to many of us whether any one physician ever sees "four hundred cases of membranous croup," or even half that number, in an average lifetime. Hence, to question this diagnosis is a natural sequence to such a report.

For myself, in a busy practice of eight years, but three cases have come under my care that were undoubted cases of membranous croup, uncomplicated with diphtheria. Fortunately, all recovered, but after many days and nights of anxiety. In one case, Ars.$^{62m}$ was the curative remedy. In another, Kali-bich. was curative, and in the other I do not remember, except to say that neither of the trio was curative. My impression is that Brom. was the remedy, but am not positive.

It is conceded that the "diagnosis" which is, in many instances, at best problematical, *suggests* to our minds *certain remedies because they are most frequently indicated*, but this cannot help us in the case at hand if the *indications* are not present for the favorite remedies.

I am confident our venerable friend would not, for the same reason, give Rhus, then Bry., then some other remedy, and then Rhus again, because these remedies have cured "between four and five hundred cases without a single failure."

Nor would he advise Bry., then Phos., then Lyc., then Bry. again for "pneumonia," for the same reasons given above. No, I must think our friend has not said what he intended, for his many valuable writings which precede do not agree with the statements made in his last productions. It will be pleasing to see a further explanation of the matter in which his former teaching is upheld.

It is unnecessary to quote from Dunham, Lippe, Guernsey, Hering, and others, for no one knows better their teaching than Dr. Wells. May it not prove to be the case that Bœnninghausen cured so many cases of the same disease with the same remedy, the cases *happening to call for that remedy,* that he stepped too far and considered the remedy almost a speeific for that disease? Does not his statement regarding Alum—in spinal paralysis—rather confirm this impression?

None will question the greatness of the man, as a physician, yet even he made mistakes, and why not profit by trying to do better?

W. S. Gee.

